# Analysis of Outcomes of the NRS 2002 in Patients Hospitalized in Nephrology Wards

**DOI:** 10.3390/nu9030287

**Published:** 2017-03-16

**Authors:** Paulina Borek, Michał Chmielewski, Sylwia Małgorzewicz, Alicja Dębska Ślizień

**Affiliations:** 1 Department of Nephrology, Transplantology and Internal Medicine, Medical University of Gdansk, 80-211 Gdansk, Poland; paulina.borek@gumed.edu.pl (P.B.); chmiel@gumed.edu.pl (M.C.); adeb@gumed.edu.pl (A.D.Ś.); 2 Department of Clinical Nutrition, Medical University of Gdansk, 80-211 Gdansk, Poland

**Keywords:** NRS-2002, nutritional status, patients with chronic kidney disease, nephrology ward

## Abstract

Introduction: Malnutrition is a common problem among hospitalized patients. In chronic kidney disease, it affects up to 50% of the population. Undernourishment has an adverse effect on prognosis and prolongs convalescence. The aim of the study was to test the effectiveness of NRS (Nutrition Risk Screening) -2002 in the assessment of risk of malnutrition for patients hospitalized in nephrology wards. The aim was to develop clinical characteristics of malnourished patients and to assess the relationship between nutritional status and patient outcome. Methods: The analysis included 292 patients, consecutively admitted to nephrology wards. NRS-2002 was assessed in comparison to subjective global assessment. Associations with patient characteristics and outcome were evaluated. Results: Out of all the respondents, 119 patients (40%) suffered from malnutrition. The NRS-2002 showed a very strong relationship with Subjective Global Assessment (SGA) (*p* < 0.0001). Malnourished patients were older, were characterized by a significantly lower body mass index (BMI), and had a much longer hospitalization duration. In multiple regression analysis, the presence of malnutrition proved to be an independent predictor of the duration of hospital stay. CONCLUSIONS: Malnutrition is highly prevalent among patients hospitalized in nephrology wards, and it affects the length of hospitalization. Identification of malnourished patients and patients at serious risk of malnutrition progression allows the implementation of appropriate nutritional intervention.

## 1. Introduction

Chronic kidney disease (CKD) is a state characterized by an impaired patient prognosis, as the mortality risk is several times higher than in the general population.

Malnutrition can be defined as any nutritional imbalance, including underweight, overweight, and nutrient deficiencies. It has a direct influence on the patient’s condition, and worsens prognosis. Malnutrition is one of the major complications of hospitalized patients, especially those suffering from chronic diseases such as CKD [1,2]. As a matter of fact, the risk of malnutrition is additionally increased in CKD patients due to dietary restrictions, and uremic toxemia resulting in the loss of appetite and aversion to food intake. Moreover, chronic low-grade inflammation—a constant complication of CKD resulting from the primary disease, uremic milieu, and/or dialysis procedure—leads to increased catabolism, further contributing to poor nutritional status.

Taking the prevalence and importance of malnutrition into account, in 2012 the national health care provider obliged all the hospitals in Poland to screen for nutritional status upon admission to a hospital ward. One of the recommended tools is NRS -2002 (Nutritional Risk Screening-2002) [3].

Given the abovementioned, the aims of our work were to evaluate the prevalence of risk for malnutrition (as assessed by the NRS-2002) in nephrology wards of a large university-based hospital, to clinically characterize malnourished patients, and to assess the relationship between nutritional status and patient outcome.

## 2. Materials and Methods

The population of the study consisted of 292 patients (M = 157), with an average age of 60 ± 17.8 years, consecutively admitted to the nephrology wards of a large university-based hospital during a six-month period. All subjects gave their informed consent for inclusion before they participated in the study. The study was approved by the university ethics committee (No. NKBBN/75/2-13). In the study population, most patients (*n* = 235; 81%) had recognized chronic kidney disease (CKD). The causes of the CKD in the study group were the following: diabetes (11.5%), glomerulonephritis (29.7%), systemic lupus erythematosus (5.1%), ischemic/hypertensive nephropathy (3.4%), pyelonephritis (13.6%), Autosomal dominant polycystic kidney disease (ADPKD, 6.4%), and other or unknown causes (30.3%).

### 2.1. Nutritional Status

Risk for malnutrition was evaluated at admission by the NRS-2002, which was applied by a trained clinical examiner. NRS-2002 consists of two parts. The first is devoted to the assessment of the patient’s nutritional status and food intake problems, while the second contains information related to the impact of disease severity on nutritional status. Each part is scored from 0 to 3 points, and patients receive an extra point for age above 70 years. In total, the patient can get 7 points; the greater the number of points, the greater the risk of malnutrition.

The results of the NRS-2002 were compared to the SGA (Subjective Global Assessment), a widely acknowledged tool for the nutritional status assessment which had been previously used on the Ward. Analysis by SGA consists of inquiries regarding the nutritional issues, loss of body weight, and the type of the diet consumed. Additionally, the patients underwent a physical examination (loss of subcutaneous tissue, loss of muscle, evaluation of edema). The grading scale in SGA is A, B, and C, where A indicates a good nutritional status and C indicates severe malnutrition [4].

Additionally, in order to assess the patient’s appetite and the risk of weight loss over the next six months, the SNAQ (Simplified Nutritional Appetite Questionnaire) was used. This quick method consists of four short questions regarding the loss of appetite, feeling of fullness, changes in taste, and the number of meals per day. The SNAQ is composed of four questions. Each question presents five options, represented by the letters from A to E. The questions are scored based on the following scale: A = 1, B = 2, C = 3, D = 4, and E = 5. When summed up, they give the total score of the SNAQ, which may range from 4 to 20. The lower the number is, the higher the risk of weight loss is. In the original instrument, indices lower than or equal to 14 indicate a risk for loss of at least 5% of body mass during the next six months [5].

In addition, each patient underwent an analysis of his/her medical records to obtain information regarding concomitant diseases. All biochemical analyses were performed using routine methods at the local laboratory. They included urea, creatinine, albumin, and C-reactive protein (CRP). Glomerular filtration rate (GFR) was estimated based on the MDRD (Modification of Diet in Renal Disease) equation. The patients were followed until the day of discharge.

### 2.2. Statistical Analysis

Results are expressed as percentages (for categorical variables), mean and standard deviation, or median and interquartile range, as appropriate. The assumption of normality was verified with the Kolmogorov–Smirnov test. A *p*-value < 0.05 was considered to be statistically significant. Comparisons between two groups were assessed with a Student’s *t*-test or a Mann–Whitney test, as appropriate. Additionally, a McNemar’s test was used to assess significant statistical relationships between the results of the assessment of NRS 2002 and SGA. Independent associations among variables were assessed with stepwise multiple regression analysis. Statistical processing of the results was performed with the use of the statistical software STATISTICA PL v 12.0 (Statsoft, Kraków, Poland).

## 3. Results

### 3.1. Nutritional Status

According to the NRS-2002, 119 (41%) of the subjects were at risk of malnutrition (NRS ≥ 3). The assessment of nutritional status by SGA showed that 120 (41%) patients were moderately or severely malnourished (received a score B or C). Analysis using the McNemar’s test showed a strong correlation between NRS 2002 and SGA (χ^2^ = 12.99; *p* = 0.003). The clinical characteristics of the studied patients with regard to their nutritional status are shown in Table 1. As expected, subjects with malnutrition had a significantly lower body mass index (BMI; 23.4 ± 6.4 vs. 25.6 ± 7.3; *p* < 0.001), serum albumin (26.0; 22.1–33.0 vs. 32.5; 25.4–36.3 g/L; *p* < 0.0001), and were older, as compared to well-nourished patients (73.0 ± 20.0 vs. 54.0 ± 22.3 years; *p* < 0.0001).

Risk for malnutrition as assessed by the NRS-2002 was associated with almost twice as long duration of hospitalization (13.0; 8.0–17.0 vs. 7.0; 6.0–14.0 days; *p* < 0.0001) (Table 1). In multiple regression analysis, the presence of malnutrition proved to be an independent predictor of the duration of hospital stay (Table 2).

To identify the difference between malnourished patients and those with normal nutritional status, the prevalence of concomitant diseases described in the two subgroups was studied. Patients with the NRS ≥ 3 and SGA score B and C suffered from cardiovascular diseases and cancer significantly more frequently than those that were adequately nourished, as shown in Table 1.

### 3.2. SNAQ

Based on the analysis of data from the SNAQ, it was found that 122 (42%) of the patients were at risk of body weight loss over the following six months. What is probably more important, 82 (69%) of malnourished patients turned out to be at a serious risk of malnutrition progression due to the lack of appetite (SNAQ ≤ 14).

**Figure 1:**
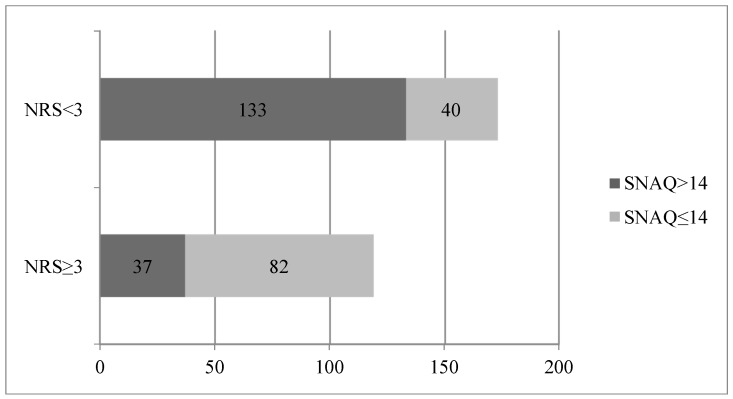
SNAQ: Simplified Nutritional Appetite Questionnaire.

In comparison, among properly nourished patients, such a risk was found in only 40 (23%) subjects (Figure 1).

### 3.3. BMI

**Figure 2:**
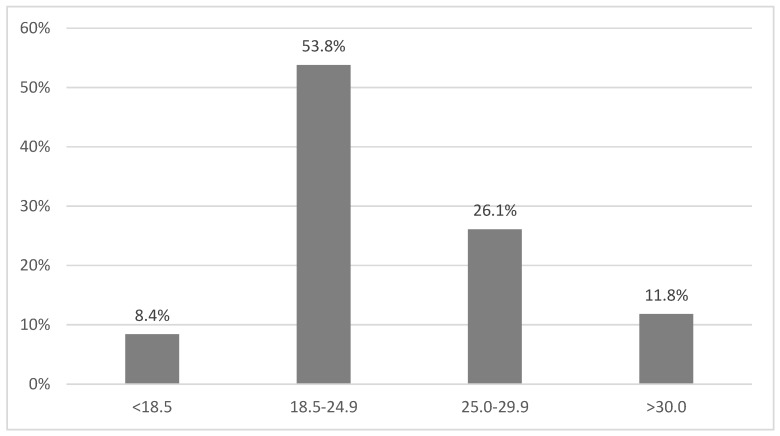
Distribution of BMI (kg/m^2^) among the malnourished patients.

Analysis of patients’ BMI showed that only 10 (8%) of malnourished patients or patients at risk for malnutrition had values below 18.5 kg/m^2^. What is more, 45 (38%) of subjects diagnosed as having malnutrition were overweight or obese (Figure 2).

### 3.4. Chronic Kidney Disease (CKD)

We analyzed the percentage of patients diagnosed with CKD and with malnutrition. The results showed a relationship between the severity of the disease as assessed by the eGFR (Estimated Glomerular Filtration Rate) and the prevalence of malnutrition. The prevalence of malnutrition increased with worsening kidney function (Table 3).

## 4. Discussion

The aim of the study was to estimate the prevalence of risk for malnutrition and malnutrition among patients treated on nephrology wards assessed with the NRS-2002, recommended by the National Health Foundation. It was evaluated in comparison to the SGA, a well-acknowledged and previously utilized tool for nutrition assessment, as well as in comparison to BMI [3,4]. Additionally, the SNAQ was analyzed, a method to assess the risk of future malnutrition development and progression [5].

The presented results confirm that malnutrition is a major public health problem among hospitalized patients. The prevalence of deterioration of nutritional status reached 41%, indicating that a considerable number of patients in nephrology wards were in need of appropriate nutritional treatment. Similar results were shown by Rasmussen et al. [6], where 40% of patients in a Danish hospital were classified as undernourished (NRS ≥ 3). Moreover, in a 2013 study conducted in internal medicine wards of Chinese hospitals, 41.5% of all examined subjects were malnourished as defined by NRS-2002 [7].

To evaluate the effectiveness of NRS-2002, we used the SGA as a reference tool for nutritional status assessment.

Our data, obtained with both methods, was similar (*n* = 119; NRS-2002 vs. *n* = 120; SGA). Risk of malnutrition in NRS-2002 and malnutrition recognized in SGA related to the same patients, as confirmed by the McNemar’s test (*p* = 0.003). However, NRS-2002 seems to be a screening method that is quick, straightforward, and useful in clinical practice.

In our study, 235 (81%) of the respondents suffered from CKD. Malnutrition was diagnosed in 91 (39%) of them. In a study by Sorensen et al. [2], the prevalence of malnutrition among CKD patients reached 29%. It is of interest that in the present study, the deterioration of nutritional status correlated well with a decrease in eGFR, and affected half of the patients in stage 5 CKD (i.e., in end-stage renal failure). One of the major reasons for malnutrition in the course of CKD is the loss of appetite, due to the growing uremic toxemia in patients with CKD treated conservatively. In the case of dialysis patients, malnutrition might additionally result from nutrient losses during dialysis, from bioincompatibility of dialysis membranes in hemodialysis, and of dialysis fluids in peritoneal dialysis. Moreover, CKD patients have a number of dietary restrictions. These include reducing the amount of ingested protein, phosphates, and potassium.

In our study, malnourished patients were statistically older (73 vs. 54 years old), as was also the case in a study by Tangvik et al. [8]. Of all the respondents, 123 (42%) were over 65 years old. Among these, 83 (68%) were diagnosed as malnourished. Previous reports indicate the prevalence of malnutrition in almost 80% of people over 65 years of age at the start of hospitalization [2,9]. In patients over 65 years of age, there is a natural reduction in the content of lean body mass. Furthermore, this group of patients is significantly more likely to suffer from diabetes, hypertension, cancer, kidney disease, peptic ulcer disease, and coronary disease, which have a significant impact on the deterioration of nutritional status [8,10]. Obviously, it has to be underlined that the patients receive one additional point for age above 70 years in the NRS-2002, introducing bias to the abovementioned results.

In our study, we also used a simple method assessing appetite (SNAQ). The data obtained showed that hospitalized people often complained of a lack of appetite or a rapid feeling of fullness. The rating of appetite seems to be especially important, because in most cases malnutrition may be due to eating inadequate portions and/or meals poor in nutrients [11,12].

Our results showed that in 42% of malnourished patients there was a risk of aggravating the current condition because of their poor appetite.

Because malnutrition affects 20%–50% of hospitalized patients, and because it has a direct effect on mortality and length of hospitalization, the detection of patients at risk of malnutrition is an essential part of clinical assessment [13,14]. The present results show that nutritional status often deteriorates during hospital stays [15], and that undernourished patients are generally hospitalized longer [16,17,18]. The impact of malnutrition on the length of hospitalization was also demonstrated in our study. The median duration of hospitalization of the undernourished patients was almost twice as long as of the subjects with normal nutritional status. Moreover, the results of multiple regression analysis showed that malnutrition was an independent factor for the prolongation of hospitalization.

Interestingly, in our study, 8.4% of patients diagnosed with malnutrition had a BMI below the normal range. In fact, 45%/38% of subjects diagnosed as having malnutrition were overweight or obese according to BMI. It seems that BMI is not sufficient as a tool to assess the nutritional status of hospitalized patients. Additionally, our previous studies show the lack of a direct relationship between BMI and malnutrition. High BMI does not exclude the presence of malnutrition in patients after kidney transplantation or with severe aortic stenosis [19,20].

The limitation of our study is the relatively small group of patients, but despite its limitation, the results of the present study are valuable because they indicate a problem of preexistent malnutrition in patients with chronic kidney disease and a risk of further deepening malnutrition (based on the SNAQ). Further studies in patients with CKD (e.g., multicenter) are needed to confirm our results. Therefore, it is necessary to pay attention to routine repeated nutritional assessment, especially in patients with CKD (e.g., every 6 months on an outpatient basis). Furthermore, the results of SNAQ indicate the need to monitor the implementation of dietary recommendations. Recommended intake may not be realized because of a poor appetite or lack of appetite.

There is the need not only to assess nutritional status upon admission, but to monitor nutritional therapy daily during hospitalization.

## 5. Conclusions/Summary

The study shows that nutritional status alterations as assessed by the NRS 2002, SGA, and SNAQ at admission to nephrology wards are a frequent finding in hospitalized patients, and that preexisting malnutrition is associated with a negative hospital outcome in these patients.

The main clinical utility of the SNAQ method is for risk stratification, because the method allows easy identification of high-risk subgroups of patients (without appetite) who probably need more aggressive nutritional intervention. 

Because most patients in the nephrology wards are at risk for malnutrition-associated morbidity and mortality, evaluation of nutritional status should become a part of both routine clinical assessment and prognostic stratification.

## Figures and Tables

**Table 1:** Patient characteristics according to Nutritional Risk Screening-2002 (NRS-2002; NRS < 3 patients not at risk, NRS ≥ 3 patients at risk for malnutrition).

Parameters	Total Sample Median (% or IQR or SD)	Nutritional Not at Risk Median (IQR)	At Risk for Malnutrition Median (IQR)
Subjects	292	173 (59.2%)	119 (40.8%)
Sex			
Male	157 (53.8%)	93 (53.8%)	64 (53.8%)
Female	135 (46.2%)	80 (46.2%)	55 (46.2%)
Age (years)	60 ± 17.8	54 (44–70) *	73 (49–74)
Length of stay (days)	12.7 (6.0–14.0)	7 (6–14) *	13 (8–17)
Body weight (kg)	71.0 ± 18.3	75 (60–85) *	65 (59–84)
Serum albumin level (g/L)	29.4 (23.0–35.0)	32.5 (25–36) *	26 (22–33)
BMI (kg/m^2^)	25.8 ± 5.8	25.6 (23–30) *	23.4 (20–28)
Disease			
Diabetes	87 (30%)	49 (28%)	38 (31%)
Chronic pulmonary obstructive disease	13 (4.4%)	7 (4%)	6 (5%)
Heart failure	75 (25.7%)	30 (17%) *	45 (38%)
Coronary heart disease	129 (44.2%)	62 (35%) *	67 (56%)
Cancer	41 (14%)	17 (10%) *	24 (20%)
Hypertension	198 (67.1%)	112 (65%)	86 (72%)

* *p* < 0.05. BMI: body mass index.

**Table 2:** Multivariate regression models predicting the duration of hospitalization (the adjusted R^2^ of the models were 0.12 and 0.19, respectively; *p* < 0.001).

Regression Models	B	Standard Error	Beta	*p*-Value
Model 1				
Constant	9.05	0.92		<0.001
NRS-2002	9.13	1.44	0.35	<0.001
Model 2				
Constant	12.7	2.87		<0.001
NRS-2002	11.78	1.64	0.45	<0.001
Age	−0.18	0.05	−0.25	<0.001
CVD	4.91	1.57	0.19	0.002
CKD	4.75	1.74	0.15	0.006

CVD—cardiovascular disease, CKD—chronic kidney disease.

**Table 3:** The prevalence of malnutrition depending on the stage of CKD.

The Value of eGFR (mL/min) (4pMDRD)	The Percentage of Patients Diagnosed with Malnutrition
>60	20%
30–59	29.2%
15–29	43.3%
<15 or the treatment of dialysis	52.2%

eGFR—Estimated Glomerular Filtration Rate); 4pMDRD—The Four-variable Modification of Diet in Renal Disease
